# Clinical Frailty Scale at presentation to the emergency department: interrater reliability and use of algorithm-assisted assessment

**DOI:** 10.1007/s41999-023-00890-y

**Published:** 2023-11-16

**Authors:** Rainer Albrecht, Tanguy Espejo, Henk B. Riedel, Søren K. Nissen, Jay Banerjee, Simon P. Conroy, Thomas Dreher-Hummel, Mikkel Brabrand, Roland Bingisser, Christian H. Nickel

**Affiliations:** 1 Emergency Department, University Hospital Basel, University of Basel, Petersgraben 2, 4031 Basel, Switzerland; 2 Research Unit for Emergency Medicine, Odense University Hospital, Odense, Denmark; 3 Department of Geriatric Medicine, Odense University Hospital, Odense, Denmark; 4 University Hospitals of Leicester NHS Trust, Leicester, UK; 5 Department of Population Health Sciences, University of Leicester, Leicester, UK; 6 St Pancras Hospital, Central and North West London NHS Foundation Trust, London, UK; 7 MRC Unit for Lifelong Health and Ageing, University College London, University College London Hospitals, London, UK

**Keywords:** Clinical Frailty Scale, Interrater reliability, Geriatric acuity, Frailty, CFS, Emergency department

## Abstract

**Purpose:**

The Clinical Frailty Scale (CFS) allows health care providers to quickly stratify older patients, to support clinical decision-making. However, few studies have evaluated the CFS interrater reliability (IRR) in Emergency Departments (EDs), and the freely available smartphone application for CFS assessment was never tested for reliability. This study aimed to evaluate the interrater reliability of the Clinical Frailty Scale (CFS) ratings between experienced and unexperienced staff (ED clinicians and a study team (ST) of medical students supported by a smartphone application to assess the CFS), and to determine the feasibility of CFS assignment in patients aged 65 or older at triage.

**Methods:**

Cross-sectional study using consecutive sampling of ED patients aged 65 or older. We compared assessments by ED clinicians (Triage Clinicians (TC) and geriatric ED trained nurses (geriED-TN)) and a study team (ST) of medical students using a smartphone application for CFS scoring. The study is registered on Clinicaltrials.gov (NCT05400707).

**Results:**

We included 1349 patients aged 65 and older. Quadratic-weighted kappa values for ordinal CFS levels showed a good IRR between TC and ST (ϰ = 0.73, 95% CI 0.69–0.76), similarly to that between TC and geriED-TN (ϰ = 0.75, 95% CI 0.66–0.82) and between the ST and geriED-TN (ϰ = 0.74, 95% CI 0.63–0.81). A CFS rating was assigned to 972 (70.2%) patients at triage.

**Conclusion:**

We found good IRR in the assessment of frailty with the CFS in different ED providers and a team using a smartphone application to support rating. A CFS assessment occurred in more than two-thirds (70.2%) of patients at triage.

**Supplementary Information:**

The online version contains supplementary material available at 10.1007/s41999-023-00890-y.

## Key summary points

**Aim:**

In this study, the interrater reliability of the Clinical Frailty Scale (CFS) ratings comparing assessments by both experienced and unexperienced staff (ED clinicians and a study team (ST) using a smartphone application to support CFS scoring) was evaluated. The feasibility of the CFS assignment at ED triage, defined as a majority of patients aged 65 or older assigned a CFS level at triage, was also investigated.

**Findings:**

In this cross-sectional study of 1349 consecutive ED patients aged 65 years and older, the interrater reliability for CFS ratings was good for three different dyads assessed, whether used as an ordinal scale or as frailty categories (CFS 1–4 = non-frail to vulnerable; CFS 5–6 = mild to moderate frailty; CFS 7–9 = severe frailty to terminally ill). More than two-thirds (70.2%) of patients had a CFS rating assigned at triage.

**Message:**

The CFS is a reliable scale for use in the ED and the implementation of frailty assessment could be facilitated with an algorithm-assisted assessment.

**Supplementary Information:**

The online version contains supplementary material available at 10.1007/s41999-023-00890-y.

## Introduction

### Background

Frailty is a state of increased vulnerability to stressors which develops as a consequence of age-related decline in several inter-related physiological systems [1, 2], and is associated with a wide range of adverse health outcomes such as delirium, falls, functional decline, prolonged hospitalization and death [3–9]. Although qualitative assessment using “clinical judgment” is often used to identify frailty, this is not a reliable method and is less accurate than using formal assessment tools [10]. Several assessment tools have been proposed to assist with the identification of frailty in the Emergency Department (ED) [10–13].

The Clinical Frailty Scale (CFS) is a 9-point scale ranging from “very fit” to “terminally ill” [14]. It is one of the most commonly used frailty assessment tools [15]. The CFS allows health care providers to quickly stratify older patients with the help of pictographs and clinical descriptors. Using the CFS in the ED may be helpful in the early identification of patients who may benefit from additional services, and to support clinical decision-making [3, 8, 9, 16]. The CFS takes less than a minute to complete and due to its simplicity and ease of use the CFS is well-suited for use in the ED [12, 17–20].

### Importance

The CFS has been investigated for interrater reliability (IRR) between nurses and physicians, between patients’ self-ratings and health care providers’ ratings, and between physicians from different specialties [19, 21–24]. In EDs, however, there has been a limited amount of studies evaluating the CFS interrater reliability, either including few patients or specific patient groups or never including nurses who specialize in care of older patients. In an ED study from the UK in which the CFS is in routine use, frailty assessment at triage was reported to include 50.3% of patients aged 65 and older [8]. A smartphone application has also been made available to aid CFS scoring in the hands of less experienced personnel. The application was developed as a response to the National Institute of Health and Care Excellence recommending use of the CFS when risk stratifying older people with COVID-19 [25].

It was previously suggested to determine frailty adjusted triage in the ED setting by assessing both acuity (with the Emergency Severity Index, ESI triage tool) and frailty (as measured with the CFS) [3, 9, 16, 26–29]. Frailty adjusted triage was implemented in our ED prior to the COVID-19 pandemic [30, 31], but to what degree this process is adhered to is unknown.

### Goals of this investigation

The primary aim of this study was to evaluate the IRR of CFS ratings comparing assessments by both experienced and unexperienced staff (ED clinicians and a study team (ST) using a smartphone application to support CFS scoring), in a consecutive sample of ED patients aged 65 and older. In addition, we aimed to determine the proportion of patients aged 65 or older who were assigned a CFS level at triage.

## Methods

### Study design and setting

This cross sectional analysis of a subgroup of patients aged 65 and older was based on a prospective observational study using consecutive sampling. All patients, aged 18 or older, who presented to the ED of the University Hospital Basel, in Northwestern Switzerland, between April 25 and May 30, 2022, were assessed for inclusion 24 h a day, 7 days a week. The University Hospital Basel is a tertiary care center with an annual ED attendance of approximately 55,000 patients aged 16 years and older, of whom about a third are aged 65 years or older. Obstetric, pediatric, and ophthalmologic patients are treated in separate facilities on campus. There are three geriatric hospitals, two palliative care facilities, and 39 nursing homes with almost 2900 nursing beds in the area.

This study is reported according to the “Guidelines for Reporting Reliability and Agreement Studies (GRRAS)” [32]. The GRRAS checklist for reporting of studies of reliability and agreement can be found in Supplemental Table 1.

### Selection of participants

This study was approved by the local Ethics Committee (EKNZ, Nr. 236/13) and conducted according to the principles of the Declaration of Helsinki. The study is registered on the Clinicaltrials.gov website (study N° NCT05400707). All patients presenting to the ED of the University Hospital Basel during the study period were screened for inclusion. Patients who were unable to provide verbal informed consent (e.g., treatment in the resuscitation bay or immediate transfer to the OR) were not included. Of note, patients with cognitive impairment who were able to provide verbal consent were not excluded to minimize bias, in accordance with previous recommendations [33, 34]. The analysis included all index visits from patients aged 65 and over.

### Data collection

During the study period (24 h a day, 7 days a week), patients were screened and interviewed by a study team member upon presentation to the ED. All data were recorded on machine-readable case report forms, which were subsequently scanned, and the data were then cleaned in a two-step process. This was carried out first, by ED administrators for handwriting issues and legibility issues, and then by an external company, Digx Gmbh®, which was also responsible for the transfer of data into the database. All other data from the patients’ Electronic Health Record (EHR) were also transferred into the database, using the unique patient ID to match it with the EHR. Demographics (such as age and sex) and the Emergency Severity Index level (ESI), a 5 level triage tool [35], were collected from the EHR. ESI triage level assignment is mandatory in all patients presenting to the ED, and can, therefore, be used as a comparator to assess adherence to frailty-adjusted score [9].

### CFS assessment

The CFS was assessed by three different groups of raters: Triage clinicians (TC), geriatric ED trained nurses (geriED-TN), and a study team (ST) of medical students supported by the CFS smartphone application [36]. The group of TC raters consisted of 67 nurses who work 24/7 and 25 triage liaison physicians who are present during highest patient influx times (i.e., during 8 a.m. and 00:00 a.m.) and are available for rapid assessments, communication and test ordering at triage [37]. The team of geriED-TN raters consisted of 8 ED nurses specialized in care of older patients with at least a bachelors’ degree. The geriED-TN are present from Monday to Friday 8 a.m. to 5 p.m. to assess patients deemed at geriatric risk. The ST group of raters consisted of medical students in their fourth to sixth year (of six years) of medical school training. The ST was unaware of the hypothesis of this study and was not involved in patient care.

As the study site is located in a German-speaking area of Switzerland, we had previously performed an authorized translation of the CFS into German with permission (Supplemental Fig. 2) [35, 38, 39]. This translation is used in clinical routine in our ED and was, therefore, used by the TC and the geriED-TN. The ST assessed the CFS using the “Clinical Frailty Scale (CFS)” application (version 1.1.0) on a handheld device [36]. The CFS smartphone application was developed by NHS Elect and clinicians in Leicester UK and specifically designed to reduce anchoring bias when using the CFS [40], by guiding users through the CFS in reverse order. This elicited negative responses to the highest level of dysfunction as opposed to settling on positive responses to the lowest degree of dysfunction thus reducing a tendency to underscore subjects.

TC, in charge of triage, i.e., determining the acuity (with the ESI), assessed the CFS during the triage process upon patients’ arrival in the ED. All patients aged 65 or older are meant to be assessed with the CFS. The geriED-TN assess CFS ratings in a subset of patients that were deemed at geriatric risk. At 8 a.m. they routinely assess these patients that stayed in the ED observation unit overnight. The ST assessed CFS in the first hour after patients’ arrival in the ED for all patients aged 65 or older.

Training of TC consisted of a day of teaching and case-based discussions in geriatric emergency medicine topics. Additionally, TC received CFS training using case vignettes. In addition to normal TC training, the geriED-TN received individual teaching by an experienced Advanced Practice Nurse, the clinical champion of the geriED-TN team while on shift. Further, every 3 months, a whole-day workshop with case-based discussions, action learning and continuing education on geriatric topics is organized. The ST received half an hour of training in the concept of frailty in general, and the CFS in particular. In addition, the members of the ST were instructed to use the application’s algorithm to determine frailty levels and used the training material provided in the application.

### Outcomes

The primary outcome was the IRR of CFS assessments between triage clinicians, a dedicated ST supported by a smartphone application, and a team of geriED-TN. As a secondary outcome, we investigated the proportion of patients that are assessed for frailty, with the CFS, in daily routine (by TC and geriED-TN). This was defined as the adherence rate. Feasibility was defined as an adherence rate in the majority of all patients, as 50% is the adherence found in a similar study [8]. In addition, the adherence rate of frailty assessments was compared to the rate of acuity assessments.

### Statistical analysis

Descriptive statistics are expressed as counts and percentages or as medians with interquartile ranges (IQR). IRR between ordinal (i.e., 1 to 9) CFS ratings was calculated using quadratic-weighted kappa statistics with 95% confidence intervals (CI) for these associations: TC – ST, TC – geriED-TN, and ST – geriED-TN. Quadratic-weighted kappa was also calculated for categorized CFS levels (CFS 1–4 = non-frail to vulnerable; CFS 5–6 = mild to moderate frailty; CFS 7–9 = severe frailty to terminally ill). A kappa value < 0.20 was considered as poor agreement, 0.20–0.40 as fair agreement, 0.41–0.60 as moderate agreement, 0.61–0.80 as good agreement and > 0.80 as very good agreement [41]. The Kappa’s 95% CI were calculated using bootstrap methods. Intraclass correlation coefficients (ICC) and their 95% confident intervals between ordinal CFS ratings were calculated based on a single rater, absolute agreement, 2-way mixed-effects model. We interpreted the ICC values as such: less than 0.5 are indicative of poor reliability, values between 0.5 and 0.75 indicate moderate reliability, values between 0.75 and 0.9 indicate good reliability, and values greater than 0.90 indicate excellent reliability [42]. Heatmaps were constructed to compare the CFS ratings between the three dyads of raters. Bland–Altman plots were constructed to illustrate the differences between two group of raters plotted against their average. All statistical analyses were performed using R, version 4.2.1 [43].

## Results

### Baseline characteristics and CFS ratings

**Fig. 1:**
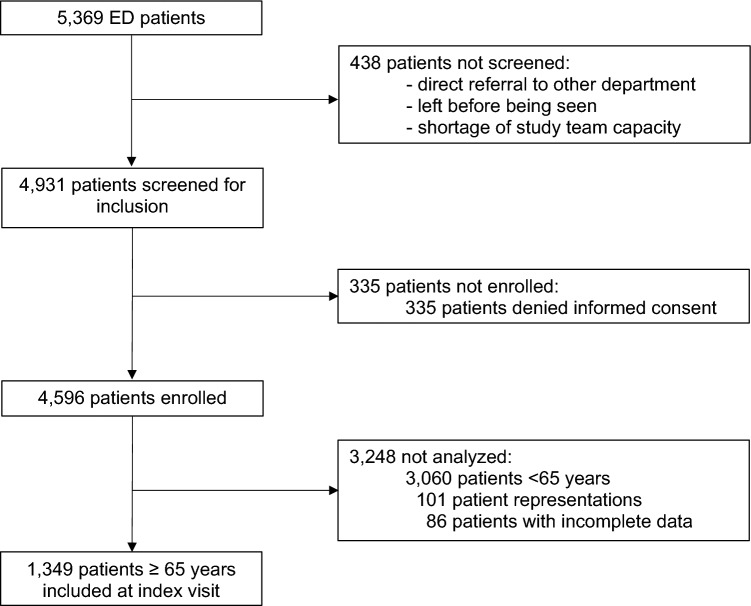
Flowchart of the study population. The chart displays the recruitment procedure of consecutive ED patients, aged 18 or older. This study report results on patients aged 65 or older. *ED* emergency department

Of 4,931 patients screened for inclusion, 335 were not enrolled because they denied informed consent. We included 1349 patients aged 65 and older of whom 708 (52.5%) were female (for details, see Fig. 1). Median age was 78 (IQR: 72, 85). A CFS level was assigned at triage in 946 patients (70.2%), of whom 342 (36.1%) were frail (CFS > 4). In comparison, an ESI level was assigned at triage in 1,342 patients (99.6%). Median CFS at triage was 4 (IQR: 3, 5). GeriED-TN assigned 131 CFS ratings to patients under their care, of whom 80 (61.1%) were frail. Median CFS assigned by GeriED-TN was 5 (IQR: 4, 6). A CFS level was assigned by the ST in 1,131 patients (83.9%), of whom 414 (36.6%) were frail. Median CFS assigned by the ST was 4 (IQR: 3, 5). Overall, 972 (72.1%) patients had CFS levels assigned by the clinical team (by either TC and/or geriED-TN). The patients who did not receive any CFS rating from the clinical team (TC and geriED-TN) were younger, were less frequently of female gender, had higher urgency (i.e., lower ESI), were more likely to be admitted to the ICU than those who were assigned a CFS by the clinical team (for details, see Table 1).

**Table 1:** Patient characteristics

	All included patients	CFS assigned by TC	CFS assigned by ST	CFS assigned by geriED-TN	CFS assigned by TC and/or geriED-TN	No CFS assigned by TC and geriED-TN
*N*, (%)	1349 (100%)	946 (70.2%)	1131 (83.9%)	131 (9.7%)	972 (72.1%)	376 (27.9%)
Age, median [IQR]	78 [72, 85]	79 [72, 86]	79 [72, 86]	82 [77, 88]	79 [73, 86]	76 [70, 84]
Female sex, *n* (%)	708 (52.5%)	524 (55.4%)	596 (52.7%)	77 (58.8%)	535 (55.0%)	173 (46.0%)
ESI, median [IQR]	3 [2, 3]	3 [2, 3]	3 [2, 3]	3 [2, 3]	3 [2, 3]	2 [2, 3]
NEWS, median [IQR]	1 [0, 2]	1 [0, 2]	1 [0, 2]	1 [0, 3]	1 [0, 2]	1 [0, 1]
Admitted, *n* (%)	820 (60.8%)	567 (59.9%)	676 (59.8%)	99 (75.6%)	582 (60.0%)	232 (63.3%)
ICU admission, *n* (%)	76 (9.3%)	37 (6.5%)	43 (6.4%)	2 (2.0%)	37 (6.4%)	39 (16.8%)
CFS, median [IQR]		4 [3, 5]	4 [3, 5]	5 [4, 6]		
CFS > 4, *n* (%)		342 (36.1%)	414 (36.6%)	80 (61.1%)		

Baseline characteristics and CFS rating for the study population, separated first by CFS assessment from each assessor and, by CFS assessment from clinical team (TC and geriED-TN) or not. Data are reported as median (IQR) or *n* (%)

*CFS* clinical frailty scale, *ST* study team, *TC* triage clinicians, *geriED-TN* geriatric Emergency Department trained nurses, *IQR* interquartile range, *ICU* intensive care unit

**Fig. 2:**
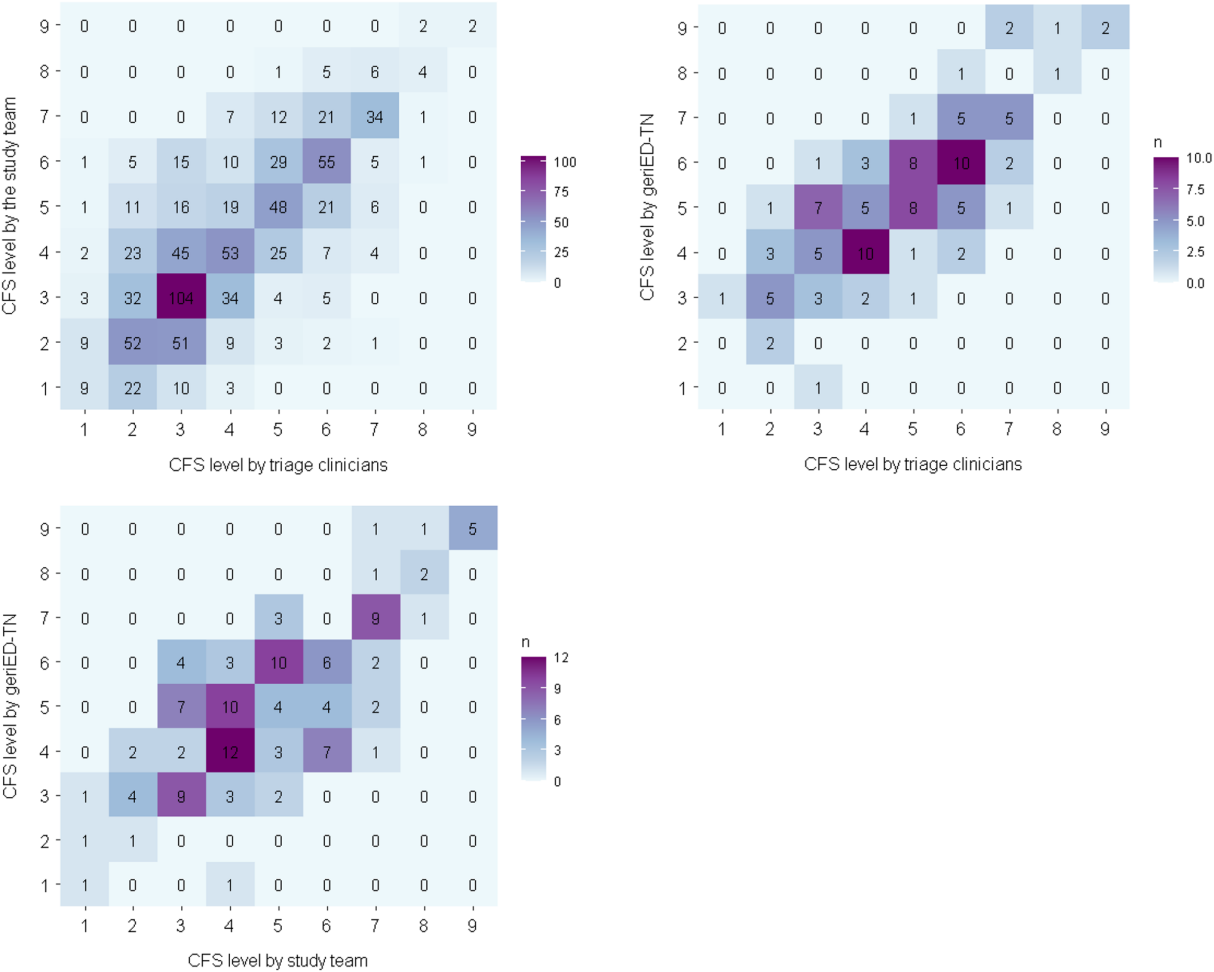
Comparison of Clinical Frailty Scale (CFS) ratings. The CFS ratings from the different assessors are on the X- and Y-axis. The number of occurrences are represented by the values in the tables and the colors. The highest proportion of patients were rated as “managing well” (CFS = 3) by the TC (27.7%) and the ST with the application (23.0%). The geriED-TN rated the highest proportion (23.0%) of patients as “mildly frail” (CFS = 5). CFS, clinical frailty scale; geriED-TN, geriatric Emergency Department trained nurses

Figure 2 displays the comparison and distribution of the CFS ratings by assessor category.

### CFS IRR

Quadratic-weighted kappa values for ordinal CFS levels showed a good IRR between TC and ST (ϰ = 0.73, 95% CI 0.69–0.76), similarly to that between TC and geriED-TN (ϰ = 0.75, 95% CI 0.66–0.82) and between the ST and geriED-TN (ϰ = 0.74, 95% CI 0.63–0.81). After categorization of the CFS ratings, the quadratric-weighted kappa values showed a good IRR for all three dyads (TC–ST: ϰ = 0.70, 95% CI 0.65–0.75 / TC–geriED-TN: ϰ = 0.70, 95% CI 0.59–0.79 / ST–geriED-TN: ϰ = 0.67, 95% CI 0.55–0.77) (for details, see Table 2).

**Table 2:** Interrater reliability of Clinical Frailty Scale (CFS) ratings

	Quadratic-weighted kappa	95% CI		*n*
		Lower bound	Upper bound	
	Between ordinal CFS (1–9)			
TC – ST	0.73	0.69	0.76	850
TC – geriED-TN	0.75	0.66	0.82	105
ST – geriED-TN	0.74	0.63	0.81	125
	Between frailty categories (CFS 1–4 = non-frail to vulnerable, CFS 5–6 = mild to moderate frailty, CFS 7–9 = severe frailty to terminally ill)			
TC – ST	0.70	0.65	0.75	850
TC – geriED-TN	0.70	0.59	0.79	105
ST – geriED-TN	0.67	0.55	0.77	125

Interrater reliability of Clinical Frailty Scale (CFS) ratings between the TC, the ST and the geriED-TN. The IRR is calculated for ordinal CFS ratings and categorized CFS ratings (CFS 1–4 = non-frail to vulnerable, CFS 5–6 = mild to moderate frailty, CFS 7–9 = severe frailty to terminally ill). Data are reported as kappa values and 95% CI. 95% CI were calculated using a bootstrap method.

*CFS* clinical frailty scale, *ST* study team, *TC* triage clinicians, *geriED-TN* geriatric Emergency Department trained nurses

The ICC showed a good reliability between TC and geriED-TN (ICC = 0.78, 95% CI 0.70–0.85) as well as between the ST and geriED-TN (ICC = 0.75, 95% CI 0.66–0.82). The ICC showed a moderate reliability between TC and the ST (ICC = 0.73, 95% CI 0.70–0.76) (for details, see Supplemental Table 2). The mean bias, from the Bland–Altman analyses, were calculated to be: −0.17 (95% CI −0.26 to −0.09) between TC and ST, −0.49 (95% CI −0.70 to −0.27) between TC and geriED-TN, and −0.27 (95% CI −0.50 to −0.05) between ST and geriED-TN. Bland Altman plots for the three dyads are shown in Supplemental Fig. 1.

## Discussion

In this cross-sectional study of 1349 consecutive ED patients aged 65 years and older the IRR of CFS ratings was good for all three different dyads assessed (TC–ST, TC–geriED-TN, and ST–geriED-TN), whether used as an ordinal scale or to categorize patients into frailty categories. In addition, we could show feasibility of the CFS at triage, as more than two-thirds (70.2%) of patients had a CFS rating assigned at triage, and in total, 72.1% of patients had a CFS rating assigned by the clinical team (TC and geriED-TN).

IRR of CFS ratings has been previously investigated. A large multi-center study demonstrated very good IRR of the CFS for a selected group of ICU patients aged 80 and older [44]. A Danish study on 40 raters that were distributed across several health care professions assessed clinical case vignettes using the Danish version of the CFS showing excellent IRR [24]. In EDs, there are limited data evaluating IRR of CFS ratings. Moderate agreement between patients’ self-ratings and health care providers’ ratings was demonstrated in North American academic EDs [19, 22]. A recent Swedish study performed in three EDs of one university hospital and two community hospitals on a total of 100 patients found moderate to good level of IRR of physicians of different specialties, registered nurses, and assistant nurses [21]. Another ED study on 100 patients compared ratings between nurses completing CFS assessments during bedside nursing to those made by one emergency physician with geriatric expertise and observed almost perfect interrater reliability [23].

Possibly, the CFS was not assessed in 402 patients (29.8%) at triage because of higher acuity (lower ESI level) and rapid treatment, e.g., in the resuscitation bay. However, this acutely-ill/-injured population is more often admitted to the ICU and could potentially benefit from frailty assessment as well, as it could help to identify the vulnerable and frail patients that are at risk of adverse outcomes, and who may benefit from extra services or care coordination [4, 5, 7]. However, the proportion of CFS ratings at triage was higher than previously described [10].

Despite the time pressure in the triage environment, the IRR of the CFS ratings was good between TC and geriED-TN. In non-concordant cases, a higher frailty level was mostly assigned by the geriED-TN. This is likely due to selection bias: geriED-TN assessed CFS in a subset of patients that were deemed to be at geriatric risk. In addition, GeriED-TNs do not have to focus primarily on identifying patients at risk for acute life-threatening events and have the opportunity to learn more about the patient and his environment. Thus, through learning more about the patient and his environment, individual aspects of frailty are more likely to be identified. Furthermore, establishment of a relation of trust may lead to more confession of deficits. This supports that assessing frailty at triage versus at disposition could be different and have a different impact [45]. While the primary function of TC is to identify patients who need to be managed quickly to avoid life-threatening events, the focus at triage is categorization of ED patients by acuity. This was highlighted by the fact that the ESI, was missing in only 6 visits during the study period, yielding to an adherence rate of 99.6%.

The questions about the degree of acceptable disagreement between raters and about the optimal use of a scale that did not show excellent reliability were raised [21]. In our study, most differences in ratings amounted to one point on this 9-point scale; more rarely, the deviation was 2 points or more. When considering frailty as a continuous entity that increases with rising CFS rating, and not a state that is present or not, this 1-point disagreement seems to be acceptable. On the other hand, if CFS ratings are used to categorize individuals as frail or non-frail and used to guide future management, a 1-point disagreement may have consequences. However, we could show that the CFS showed a good IRR even when used to categorize patients in frailty categories.

The implementation of an assessment tool in clinical practice is inevitably accompanied by challenges, particularly due to the additional workload [13]. Skills and beliefs about capacities were identified to be another possible barrier to implementing an assessment tool [46]. In addition, the use of an algorithm to assist assessment of the CFS was shown to help with reliable scoring by untrained raters [47]. We should ideally aim for caregivers with experience and training in the concepts of geriatric emergency medicine and not for unexperienced staff without specific knowledge in these areas. However, allowing less experienced staff to assess frailty with an algorithm could facilitate the implementation of frailty screening. Barriers that hindered people using frailty tools were identified to be the feeling that “it is not a priority” or that “it is someone else’s role” [10, 48] and allowing any caregiver to assess the CFS would help avoid these barriers. Incorporating frailty into the broader management plan may become more and more standard in the future. A routine, rapid and reliable standard assessment of frailty at arrival may be helpful for implementation.

## Limitations

This was a single-center study in Switzerland, focused on a mostly Caucasian population, with inclusions during a 5-week period from April to May. The results of this study may not be generalizable to other hospitals, populations, or times of year. A certain inclusion bias can be assumed as the patients aged 65 and older that did not had a CFS assigned from the clinical team were younger and of higher urgency than those who did receive a CFS rating. By excluding non-consenting older adults for ethical concerns, we may have excluded frail patients and this could have impacted our results [33]. As there was no direct comparison (medical students scoring CFS without support by a smartphone application), it cannot be concluded for sure that the support by a smartphone application for CFS scoring improved interrater reliability. We did not record the information available on the pre-admission status of the patients when assessing the CFS, and it possible that the three groups of raters did not had the same information at time of assessment.

The groups are described in aggregate and no sub-analysis within groups were performed. We cannot assure that there is no variation of reliability within groups.

We used version 1.2 of the CFS tool. In the meantime, the CFS was updated to version 2.0 to address a caveat that assessment of the habitual health state of patients rather than the state of acute illness should occur. To help with this, level headings were revised in version 2.0. As these are minor differences between the versions, we speculate that the results of this study will still be applicable to CFS 2.0 [49].

## Conclusion

With an increasingly aging population, there is a need to consider the aspect of frailty in older people during clinical assessment, so that clinical treatment concepts can be adapted accordingly. We found good IRR in the assessment of frailty with the CFS in different ED providers and a team of medical students using a smartphone application to support rating. Therefore, the implementation of frailty assessment could be facilitated with an electronic decision support. In our ED we demonstrated feasibility of scoring the CFS at ED triage, as CFS assessment occurred in more than two-thirds (70.2%) of patients at triage.

### Supplementary Information

Below is the link to the electronic supplementary material.

Supplementary file1 (DOCX 136 KB)

## Data Availability

The data that support the findings of this study are available from the corresponding author upon reasonable request, supported with a letter of authorization from an ethics committee.
