# Supplementary material for: Clinical Frailty Scale at presentation to the emergency department: interrater reliability and use of algorithm-assisted assessment
Source: Eur Geriatr Med. 2023 Nov 16;15(1):105–13. doi: 10.1007/s41999-023-00890-y (PMC10876739; doi:10.1007/s41999-023-00890-y)
Supplement: Supplementary file 1 — Supplementary file1 (DOCX 136 KB) [file 41999_2023_890_MOESM1_ESM.docx]

**Clinical Frailty Scale at Presentation to the Emergency Department: Interrater Reliability and Use of Electronic Decision Support**

**Authors List:** Rainer Albrecht^1^, Tanguy Espejo^1^ (shared first author), Henk B. Riedel^1^, Søren K. Nissen PhD^2,3^, Jay Banerjee MD^4,5^, Simon P. Conroy MD^6,7^, Thomas Dreher-Hummel^1^, Mikkel Brabrand MD^2^, Roland Bingisser MD^1^, and Christian H. Nickel MD^1^

**Affiliations:**

^1^ Emergency Department, University Hospital Basel, University of Basel, Basel, Switzerland

^2^ Research Unit for Emergency Medicine, Odense University Hospital, Odense

^3^ Department of Geriatric Medicine, Odense University Hospital, Odense

^4^ University Hospitals of Leicester NHS Trust, Leicester, UK

^5^ Department of Population Health Sciences, University of Leicester, Leicester, UK

^6^ St Pancras Hospital, Central and North West London NHS Foundation Trust, London, UK

^7^ MRC Unit for Lifelong Health and Ageing, University College London, University College London Hospitals, London, UK

**Corresponding Author**

Christian H. Nickel, Emergency Department, University Hospital Basel, Petersgraben 2, CH-4031 Basel, Switzerland

Email: [christian.nickel@usb.ch](mailto:christian.nickel@usb.ch); Twitter: @replynickel

Phone: +41 61 265 58 30

**Suplemental Table 1:** GRRAS checklist for reporting of studies of reliability and agreement

*Version based on Table I in: Kottner J, Audigé L, Brorson S, Donner A, Gajeweski BJ, Hróbjartsson A, Robersts C, Shoukri M, Streiner DL. Guidelines for reporting reliability and agreement studies (GRRAS) were proposed. J Clin Epidemiol. 2011;64(1):96-106*

| **Section** | **Item #** | **Checklist item** | **Reported in:** |
| --- | --- | --- | --- |
| Title/Abstract | 1 | Identify in title or abstract that interrater/intrarater reliability or agreement was investigated. | Title and abstract |
| Introduction | 2 | Name and describe the diagnostic or measurement device of interest explicitly. | Section 1.1 |
|  | 3 | Specify the subject population of interest. | Section 1.3 |
|  | 4 | Specify the rater population of interest (if applicable). | Section 1.3 |
|  | 5 | Describe what is already known about reliability and  agreement and provide a rationale for the study (if applicable). | Section 1.2 |
| Methods | 6 | Explain how the sample size was chosen. State the determined number of raters, subjects/objects, and replicate observations. | Section 2.2  and 2.4 |
|  | 7 | Describe the sampling method. | Section 2.2 |
|  | 8 | Describe the measurement/rating process (e.g. time interval between repeated measurements, availability  of clinical information, blinding). | Section 2.4 |
|  | 9 | State whether measurements/ratings were conducted independently. | Section 2.4 |
|  | 10 | Describe the statistical analysis. | Section 2.6 |
| Results | 11 | State the actual number of raters and subjects/objects  which were included and the number of replicate observations which were conducted. | Section 3.1 |
|  | 12 | Describe the sample characteristics of raters and subjects (e.g. training, experience). | Section 3.1 |
|  | 13 | Report estimates of reliability and agreement including measures of statistical uncertainty. | Section 3.2 |
| Discussion | 14 | Discuss the practical relevance of results. | Section 4 |
| Auxiliary material | 15 | Provide detailed results if possible (e.g. online). | Supplemental material |

**
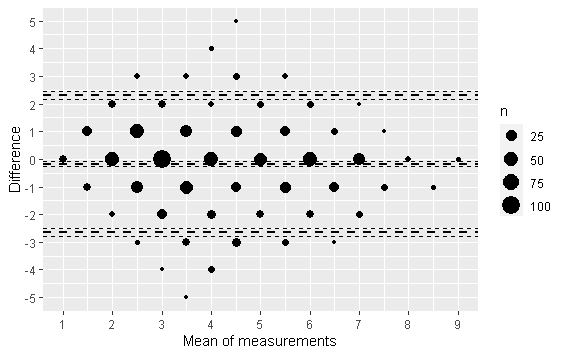
**

**Triage clinicians – study team**

**Triage clinicians – geriED-TN**

**Suplemental Fig. 1** Bland Altman plots


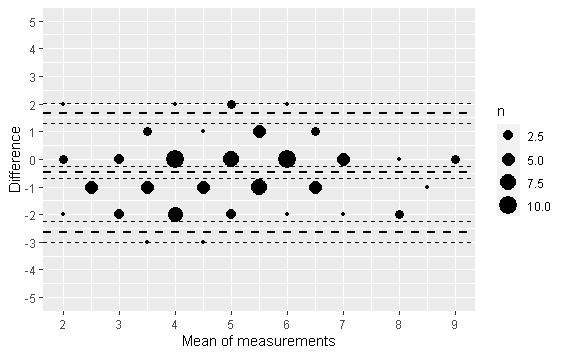


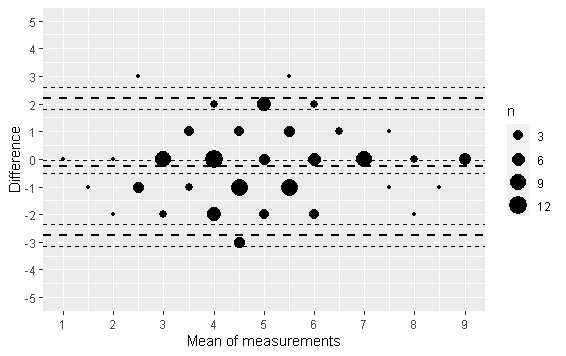


*Legend: Bland Altman plot with differences between the CFS ratings on the Y-axis and the average of these CFS ratings on the X-axis. Mean bias and limits of agreements are displayed with their 95% CI*

*Abbreviations: geriED-TN, geriatric Emergency Department trained nurses; CI, confidence interval*

**
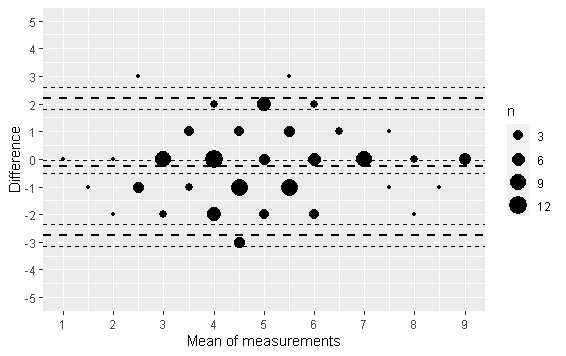
**

**GeriED-TN – study team**

**GeriED-TN – study team**

**Suplemental Table 2**: Intraclass correlation coefficients (ICC)

|  | **ICC**  *between ordinal CFS (1-9)* | **95% CI** | | ***n*** |
| --- | --- | --- | --- | --- |
|  |  | *Lower bound* | *Upper bound* |  |
| **TC – ST** | 0.73 | 0.70 | 0.76 | *850* |
| **TC – geriED-TN** | 0.78 | 0.70 | 0.85 | *105* |
| **ST – geriED-TN** | 0.75 | 0.66 | 0.82 | *125* |
|  |  |  |  |  |
| **TC – ST – geriED-TN** | 0.70 | 0.61 | 0.78 | *99* |

*Legend: ICC (two-way mixed-effects model, single rater, absolute agreement) and 95% CI between the triage, the study team and the geriatric care team.*

*Abbreviations: ICC, intraclass correlation coefficients; CFS, clinical frailty scale; TC, triage clinicians; geriED-TN: geriatric Emergency Department trained nurses.*

**Supplemental Fig. 2** German version of Clinical Frailty Scale (CFS)

*LegenThe Clinical Frailty Scale (CFS) translated in German with original pictograms*

*Abbreviations: CFS, clinical frailty scale.*
